# Combination of conditional random field with a rule based method in the extraction of PICO elements

**DOI:** 10.1186/s12911-018-0699-2

**Published:** 2018-12-04

**Authors:** Samir Chabou, Michal Iglewski

**Affiliations:** Computer Science and Engineering Department, Université du Québec en Outaouais, Gatineau, J8Y 3G5 Canada

**Keywords:** PICO, MLMs, CRF, RBMs, Information extraction, NLP

## Abstract

**Background:**

Extracting primary care information in terms of Patient/Problem, Intervention, Comparison and Outcome, known as PICO elements, is difficult as the volume of medical information expands and the health semantics is complex to capture it from unstructured information. The combination of the machine learning methods (MLMs) with rule based methods (RBMs) could facilitate and improve the PICO extraction. This paper studies the PICO elements extraction methods. The goal is to combine the MLMs with the RBMs to extract PICO elements in medical papers to facilitate answering clinical questions formulated with the PICO framework.

**Methods:**

First, we analyze the aspects of the MLM model that influence the quality of the PICO elements extraction. Secondly, we combine the MLM approach with the RBMs in order to improve the PICO elements retrieval process. To conduct our experiments, we use a corpus of 1000 abstracts.

**Results:**

We obtain an F-score of 80% for P element, 64% for the I element and 92% for the O element. Given the nature of the used training corpus where P and I elements represent respectively only 6.5 and 5.8% of total sentences, the results are competitive with previously published ones.

**Conclusions:**

Our study of the PICO element extraction shows that the task is very challenging. The MLMs tend to have an acceptable precision rate but they have a low recall rate when the corpus is not representative. The RBMs backed up the MLMs to increase the recall rate and consequently the combination of the two methods gave better results.

## Background

One of the keys to successful information extraction in the medical domain is to define the clinical question as accurate as possible. A question in natural language without any structure is very difficult to analyse. Evidence-Based Medicine (EBM) [1] is a widely-accepted methodology for medical practice that emphasises the importance of evidence from patient-centered clinical research in the health care process. EBM suggests that a clinical question should be structured in terms of four anatomic parts: patient problem or population (P), intervention (I), comparison (C) and outcome (O). These anatomic parts, known as the PICO elements, facilitate the search for answers to clinical questions.

Even though there was some controversy around the use of PICO [2], there is a consensus that the PICO is a good framework for clinical questions and some authors [3] have proven that these elements are frequently existent in medical abstracts. Formulating a question compliant with the PICO framework facilitates the search for answers to clinical questions. However, extracting PICO elements from non-structured information such as a collection of medical abstracts is challenging task. The trend is to use the machine learning methods (MLMs), known for their robustness, to extract PICO elements rather than rule based methods (RBMs). In this paper, we propose a novel approach that combines the MLM methods and the RBM methods to optimize the extraction of PICO elements within medical abstracts. Our MLM method is designed after the analysis that we carried on the aspects that influence the quality of the PICO elements extraction. Our RBM method relies on rules that use the MLM features to facilitate the integration of the both methods.

## Related work

There is a significant body of research on extracting PICO elements from abstracts of clinical documents , [3–12]. The recent trend is toward using machine-learning methods that apply a statistical model to classify sentences according to PICO framework [2]; this trend is motivated by the robustness of the MLMs and their high degree of learning.

The accuracy of the PICO statistical model depends heavily on the quality of the training corpus. Though it is difficult to specify the minimal quality requirements, we consider that most of the training corpora used in the literature are either not representative in terms of size [8, 10, 13] or not well balanced in terms of:

- the distribution of PICO elements [11, 12, 14] or
- the abstract types (structured, unstructured) [5–7, 9]

Table 1 shows an overview of the corpora used in the literature; the training corpus is usually built manually by medical experts who label the training corpus with different PICO elements. A corpus is mixed when it contains a mixture of structured and unstructured abstracts.

**Table 1:** Literature review summary of used corpora

Reference	Training Corpus	Testing Corpus
[8]	275 Manual and mixed	358 Mixed
[10]	148 Manual and mixed	75 Mixed
[13]	50 Manual and mixed	156 Mixed
[12]	800 Manual and mixed	200 Mixed
[11, 14, 30, 31]	1000 Manual and mixed	200 Mixed
[9]	1575 to 2280 Automatic and only structured abstracts	318 Mixed
[5–7]	2394 to 14,279 Automatic and only structured abstracts	2394 to 14,279 Only structured

The sizes of the corpora used in [8, 10, 13] are small and it is difficult to generalize these results. In [11, 12, 14] the distribution of PICO elements is not balanced; the P element sentences represent only 6.8%, whereas the I sentences are only 5.8%; the O sentences are more dominant with 36.6%. Such a distribution has a significant impact on the recall rate because the model did not learn enough about P and I elements. In [5–7] and [9], the authors got around the difficulty of constructing manually a large training corpus. They used the information encapsulated in MEDLINE structured abstracts that contain headings corresponding to the PICO elements. In this case, we do not have to depend on an expert of the medical domain, but we restrict the learning process to certain headings. Recently [4] proposed a novel approach for PICO extraction based on an improved Distant Supervision [15, 16]. The learning model is based on a big structured database (Cochrane), lots of unstructured data and a small amount of manually labeled unstructured data used to reduce the noise in distantly derived annotations. Notably, their Supervised Distant Supervision model automatically extracts PICO sentences from full texts compared to the literature review where the PICO extraction was limited to paper abstracts.

Most of the researches on PICO element extraction with MLMs use a non-realistic data collection, consequently the extraction performance is affected and the results are not consistent. For example, some researches state that the usage of medical semantics features is useful [7, 8, 17] while others deny the pertinence of semantic features [12, 14]. In addition, the proposed MLM methods perform inadequately with unstructured abstracts.

Generally, most of these researchers reported a precision over 70% (Table 2); however, we observed that the recall measure is usually not as high as the precision, especially when the training corpus is unbalanced in terms of PICO elements or the MLM features are not rigorous enough.

**Table 2:** Examples of reported precisions and recalls from review of the literature

	Population		Intervention	
Ref.	Precision %	Recall %	Precision %	Recall %
[9]	56–77	37–40	77–87	71–80
[13]	NA	NA	76–89	58–65
[17]	70	24	74-78	56-58
[10]	97	74	NA	NA
[7]	66-94	61-84	50-79	26–65

In order to reduce the impact of the unavailability of a representative and balanced corpus and the lack of well-designed MLM aspects, we propose a PICO element extraction system based on:

1. a MLM (CRF [18]) with well-designed aspects, these aspects include CRF parameters setting, information redundancy, type of feature value, features concordance, standardization of the abstract structure,
2. a new set of RBM rules based on the MLM features to facilitate the integration of the two methods. RBMs can have a high degree of PICO element coverage; therefore, they can complement the MLMs to improve the recall rate,
3. a hybrid combination of MLMs and RBMs. Some authors suggested the combination of the two methods. In [8], the authors extract the I and P elements using a set of RBMs that rely heavily on the UMLS concepts while they use MLMs to extract the O element because the O element does not have corresponding UMLS concept and makes it difficult to craft an efficient extracting rule. In [19], the authors use the two methods to extract the key characteristics of clinical trials from full-text journal articles reporting on RCTs. In a first stage, they use an MLM based on SVM algorithm to locate the sentences that have the highest probability of describing a trial characteristic; in the second stage, they apply simple rules to these sentences to extract text fragments containing the target answer. In our case, we complement the MLM method with RBMs to extract PICO elements. We take advantage of the robustness of the MLM method to extract the majority of the potential PICO sentences (coarse-grained), then we apply a set of RBM rules (fine-grained) designed with MLM features to extract the PICO sentences that are missed by the MLM stage.
4. cTAKES (Apache clinical Text Analysis and Knowledge Extraction System) medical pipeline [20]. cTAKES is an open source natural language processing system for information extraction from clinical natural text. It provides a type system based on the Clinical Element Model (CEM) [21] that targets and facilitates the deep semantics of the medical field. For example, it can identify the clinical named entities from various dictionaries including the UMLS.

The proposed system improves the PICO extraction process and facilitates the validity of the answers to clinical questions formulated with the PICO framework.

## Methods

First, we conduct several experiments to analyze the aspects that influence the generation of a CRF model and improve them in order to enhance the quality of the predictions generated by the model. This analysis is even more relevant when we are constrained with the unavailability of a balanced and representative training corpus.

Secondly, we propose a set of RBM rules based on the MLM features to facilitate the integration of the two methods.

Finally, we combine MLMs and RBMs to carry out a novel hybrid approach in two iterations. In the first iteration, we apply an MLM method to take advantage of the robustness of the MLMs. In the second iteration, we apply an RBM method on the abstracts that failed the MLM labeling process in order to refine the results.

### Experiments data

To validate our results we have used the same corpus as in [12] and [11]. This corpus was extracted by the authors of [14] from MEDLINE, in order to extract relevant abstracts; the authors used queries from two institutions that develop systematic reviews of the literature: The Global Evidence Mapping Initiatives (GEM) [22] and The Agency for Healthcare Research and Quality (AHRQ) [23]. A corpus of 1000 abstracts was extracted; to assure the diversity, 500 of them were randomly selected from a list of queries available in the GEM, the other 500 abstracts were randomly sampled from another set of AHRQ queries.

The 1000 abstracts were annotated manually by a medical student with the collaboration of a senior medical expert [14].

We used the same subset of abstracts as in [12] and [11] for the training and testing:

- The training set consists of 800 abstracts of which 486 are unstructured and 314 are structured.
- The test set consists of 200 abstracts of which 120 are unstructured and 80 structured.

We evaluated our MLM model using the information retrieval (IR) measures Precision (P), Recall (R) and F-Score defined as follows:

$$ \mathrm{Precision}=\mathrm{TP}/\left(\mathrm{TP}+\mathrm{FP}\right) $$

$$ \mathrm{Recall}=\mathrm{TP}/\left(\mathrm{TP}+\mathrm{FN}\right) $$

$$ \mathrm{F}-\mathrm{score}={2}^{\ast }\ {\mathrm{P}}^{\ast }\ \mathrm{R}/\left(\mathrm{P}+\mathrm{R}\right) $$

where TP means *true positive*, FP *false positive*, and FN *false negative*.

The distribution of PICO elements (Table 3) in this corpus is not balanced. There is a low number of sentences labeled with P and I compared to sentences labeled with Outcome, Other or Background. In this context, it becomes challenging to create a CRF model that enables us to predict adequately I and P sentences. Even more, the presence of the Background label adds noise to the training data; in fact, Background sentences could overlap with Population and Intervention sentences.

**Table 3:** Training corpus analysis

Label	Number of sentences	%
Population	662	6.8%
Intervention	565	5.8%
Outcome	3564	36.6%
Other	2712	27.9%
Study Design	193	2.0%
Background	2031	20.9%
Total	9727	100.0%

### CRF (MLM) stage

In this section, we describe our PICO element extraction system; the system is based on well-designed CRF aspects.

#### System description

**Fig. 1:**
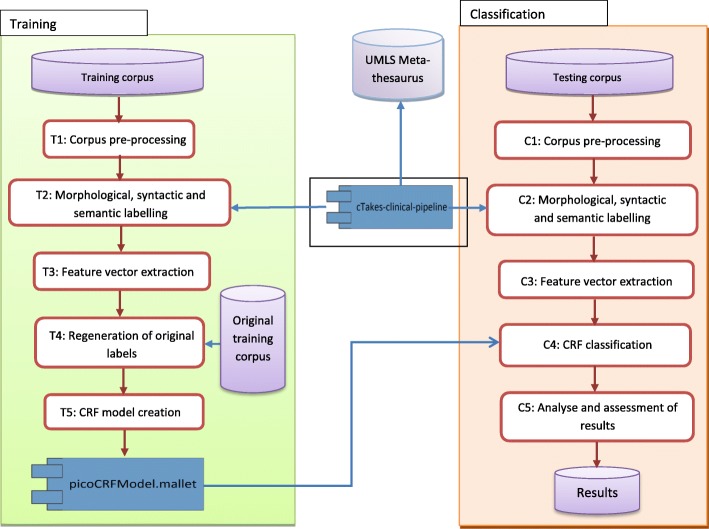
PICO element extraction system

The system is composed of a training phase and a classification phase (Fig. 1).

**T1: Corpus pre-processing** rectifies or eliminates irregularities that may exist either in the content of abstracts or in the implementation of some cTAKES classes (segmentation and POS). For example, it

- removes the characters that can be confused with the end of sentence such as vs., %, E.g.,
- corrects invalid decimal point numbers that cTAKES could consider as the end of sentence,
- standardizes section headers.

These irregularities can greatly affect the quality of PICO element extraction.

**T2: Morphological, syntactic and semantic labelling** recognizes sentences, POS tags, canonical form of word, UMLS semantic tags, medical entities, predicate-argument relations, the context of the medical entity, etc. We use cTAKES medical pipeline to achieve this task.

**T3: Feature vector extraction** extracts the various feature values to generate feature vectors, one vector by sentence. We distinguish three types of features: semantic, structural and lexical (Table 4).

**Table 4:** Types of features

Semantic features	
*f*_1_	Number of words in the sentence that are in the age, race or gender keywords list
*f*_2_	Number of words belonging to the UMLS semantic group «Disorders»
*f*_3_	Number of words belonging to the UMLS semantic group «Procedures» or «Chemicals & Drugs»
*f*_4_	Number of words that are in the Outcome keywords list
Structural features	
*f*_5_	Number of words of the sentence that are in the title
*f*_6_	Number of words of the sentence that are in the abstract’s « keywords »
*f*_7_	Sentence header
*f*_8_	Sentence length (number of words)
*f*_9_	Sentence relative position
Lexical feature	
*f*_10_	The current word and its POS belongs to the bag-of-words

**T4: Regeneration of original labels** finalizes the creation of the training corpus that is based on the 10 features (semantic, structural and lexical). T4 reuses the labels of the original training corpus [12].

**T5: CRF model creation** generates the CRF model. CRFs assign the sequence of the most probable labels Y to a sequence of observations X using the conditional probability P (Y | X) which takes the form [18]:

*P(Y|X) =* $$ \frac{1}{Z_x} $$ *exp (* $$ \sum \limits_{t=1}^T{\sum}_k{\lambda}_k{f}_k\left({y}_{t-1},{y}_t,x,t\right) $$ *).*

and can be expressed as: given an input sentence (X), what is the probability that this sentence would be classified as a PICO element (Y); Y=P, I, C or O; ʎ_*k*_ is a weight associated with the feature *f*_*k*_ and it captures how closely the given feature function is related to the given label Y. The set of weights ʎ_*k*_ represents the CRF model. T5 uses Mallet [18] to generate the CRF model based on the values of *f*_*k*_ which are stored in the feature vector.

The choice of the CRF algorithm is motivated by the fact that CRFs perform well for sentence classification with PICO labels ([9, 12, 14]). We also choose CRFs in order to validate our results against the results of [12] and [11].

The classification phase in the system architecture (Fig. 1) has the same steps as the training phase with the exception of C4 and C5.

**C4: CRF classification** applies the CRF model on the test corpus to classify sentences according to PICO elements. C4 calculates the conditional probability P (Y | X) on each sentence previously converted to a feature vector. This step uses the model picoCRFModel.mallet in the Mallet environment to predict the PICO labels of the sentences.

**C5: PICO element assessment and selection** identifies the most potential sentence for each PICO element. At the classification phase (C4), different sentences can be classified under the same PICO element, e.g. element P. We need to assess the pertinence of each sentence that competes for the same PICO element. In the review of literature some of the authors have only used the positional aspect as a main criterion [5, 8, 24]; others have used a baseline [9, 25], cross-validation [14, 17] or voting between many MLM classifier [7]. In our case, we suggest some rules to assess the pertinence of the sentence against the PICO elements. These rules are based on the positional features, the semantic features and the coexistence of different PICO elements in the same phrase. For example, we define the following rule to assess the most potential sentence for the P element:

$$ \mathrm{wTotalPopulation}=\mathrm{wPoM}+\mathrm{wPoT}+\mathrm{wCo}+\mathrm{wSe} $$

• wPoM is a positional weight which depends on the positional feature f_7_:

if f7 = Method, then wPoM = 2;

otherwise, wPoM = 0.

In the case of unstructured abstracts:

if f_7_ is in the first third of the abstract, then wPoM = 2; otherwise, wPoM = 0.

The P sentences are generally placed in the Method section (or the first third of the abstract); hence, we used arbitrary weights of 2 and 0 to favour the sentences in the Method section over the others placed elsewhere. The choice of 2 and 0 is to slightly favour the position in the Method section over the others without marginalizing this position by assigning to it a higher weight. Similar reasoning is used in [8] and the authors [5] and [24] have concluded in their research that PICO element are potentially located in the first third or the last third of the abstract.

• wPoT is another positional weight and is equal to the value of feature f_5_. The bigger f_5_ is, the richer is the sentence with the semantics of the P element therefore the more likely the sentence is to represent this element.

• wCo is a weight that depends on the coexistence of the P and I elements in the same sentence.

If P and I coexist, then wCo = 2;

otherwise, wCo = 0.

As in the case of wPoM, 0 and 2 are arbitrary weights to favour sentence that cover both elements: P and I

- wSe is a weight which depends on the presence of semantic features of the P element in the sentence: wSe = f_1_ + f_2_.

Similar rules are defined for the I and O elements. For I:

$$ \mathrm{wTotalIntervention}=\mathrm{wPoM}+\mathrm{wPoT}+\mathrm{wCo}+\mathrm{wSe} $$

wSe, in this case, is equal to the semantic feature f_3_.

We use a similar rule for the O element.

We sort the P, I and O sentences by their total weight and select the sentence with the highest weight respectively.

#### Analysis of MLM aspects

Our objective is to analyze the aspects that could influence the CRF model performance. This analysis is even more useful when CRF is trained with a non-representative corpus. To validate our results, we use the same training and test corpus as in [12].

##### Experiments setting

Given the non-balanced nature of the corpus, we have conducted many experiments to analyze the aspects that affect the CRF.

These experiments allow us to discover which experiment gives the best F-scores. They are crucial for us to study the aspects that can influence the quality of a CRF model and to suggest their improvements. We consider the following aspects:

- Model setting: Gaussian prior and training-proportion parameters
- Training information layout: standard structure vs. information redundancy structure
- Mixing different features
- Type of feature values: binary vs. natural vs. categorical
- Standardisation or not of section headings
- Grouping structural features vs. non grouping
- Mixed abstracts vs. only structured ones
- Balancing of PICO element distribution

These aspects are mutually independent; a set (1, 2 or 3) of experiments for each aspect is sufficient to evaluate the different choices. For example, to evaluate the aspect of «type of feature values», we conduct three experiments, one for each type of values: binary, natural and categorical.

We iterate over all aspects. We identify the impact of each one of them on the CRF model and we choose the best value.

In each experiment, we use the corpus of 1000 abstracts and the same subset for the training and testing as in [12] and [11]. We use Mallet [18] to train the CRF model.

We generate four sets of results:

- the MLM results, so we can validate our CRF model with one of those used in [12] and [11],
- the RBM results to analyse how the RBM stage performed on the abstracts that are not labelled by the MLM stage,
- the combined MLM and RBM results to compare them with the results in the literature review,
- the 5-fold cross validation to assess overfitting and robustness of the model.

##### Model setting

We set the CRF model with different values of the Gaussian prior such as 0.1, 1, 10, and 100. We have obtained the best results with a variance value of 10. We found that the Gaussian prior value is influenced by the quality of the training corpus; the higher the quality of the training corpus, the more advantageous to generate the model with low Gaussian prior is; this means that the model can predict reliable PICO labels that do not deviate much from the exact labels. However, as described in the Section 3.1, the training corpus does not have a good distribution of PICO elements (Table 3), and some of the annotations are inconsistent. For these reasons, we adjusted the choice of the Gaussian prior to the quality of the training corpus. We set the Gaussian prior to an average of 10 to allow various possible predictions and we repeated 3 times every sentence in the test file to increase the chances that a good prediction is among one of three repetitions. The choice of three repetitions is motivated by the number of classes of interest which are P, I and O.

The training-proportion parameters of Mallet can be set to different values; by default, they are set to 50% for training and 50% for testing. Since we will test the model with an external test corpus, we set the training proportion to 100% in order to let Mallet train the model on the full training set.

Table 5 shows how the CRF model operates on the test file to predict the sentence labels.

**Table 5:** Label prediction by the CRF model on the test file

Sentence	Conditional probability calculated by the FRC model	Sentence label
1	P (POPULATION | Phrase1) = p1	p4 > p1, p2, p3 ➔label = OTHER
	P (INTERVENTION | Phrase1) = p2	
	P (OUTCOME | Phrase1) = p3	
	P (OTHER | Phrase1) = p4	
1	P (POPULATION | Phrase1) = p1	p2 > p1, p4, p3 ➔label = INTERVENTION
	P (INTERVENTION | Phrase1) = p2	
	P (OUTCOME | Phrase1) = p3	
	P (OTHER | Phrase1) = p4	
1	P (POPULATION | Phrase1) = p1	p1 > p2, p4, p3 ➔label = POPULATION
	P (INTERVENTION | Phrase1) = p2	
	P (OUTCOME | Phrase1) = p3	
	P (OTHER | Phrase1) = p4	

This setting has improved the results of PICO extraction and facilitated the detection of cases when a sentence can be classified with multiple labels (P and I for example).

##### Training information layout

We compared the method that repeats the entries with the standard method without duplications (Table 6). We evaluated the effects of information redundancy that some authors [18] propose as a means to improve the model.

**Table 6:** Training information layout

Training file with information redundancy layout					
Sentence	Features			Label	Prediction
S1	f_1_	f_2_	f_3_	INTERVENTION	0
S1	f_1_	f_2_	f_3_	POPULATION	1
S1	f_1_	f_2_	f_3_	OUTCOME	0
S1	f_1_	f_2_	f_3_	OTHER	0
S2	f_1_	f_2_	f_3_	INTERVENTION	1
S2	f_1_	f_2_	f_3_	POPULATION	1
S2	f_1_	f_2_	f_3_	OUTCOME	0
S2	f_1_	f_2_	f_3_	OTHER	0
Training file standard layout					
Sentence	Features				Label
S1	f_1_	f_2_	f_3_	…	POPULATION
S2	f_1_	f_2_	f_3_	…	INTERVENTION
S2	f_1_	f_2_	f_3_	…	POPULATION

In the training file with information redundancy, each entry contains the features, the label and the prediction of the sentence. The sentence is classified for each of the possible labels. The example shows that the sentence S1 is a POPULATION sentence since the label prediction value is 1, contrary to 0 indicating that the sentence is not qualified for the corresponding label. The information redundancy method did not give the best results.

##### Mixing different features

We made several tests to assess the impact of the feature choice. For example, we combined structural features with lexical features, semantic features with lexical features and the three types of features together. The combination of the three features trained better the CRF model and allowed it to capture how closely a given sentence is related to a PICO element.

##### Type of feature values

Unlike the other researchers who use either binary or natural values of features, we suggest using semantic features with categorical values:

- MPt category represents the characteristics of the Patient element like “patient”, “age”, “adult”, etc.
- MP category represents the characteristics of the Problem element belonging to a UMLS semantic type such as *Gold Syndrome Disease*, *Injury or Poisoning*, *Anatomical Abnormality*, etc.
- MI category represents the characteristics of the Intervention element belonging to a UMLS semantic type like *Procedures*, *Chemicals and Drugs*, *Devices*, etc.
- MT category contains the words of the title of the abstract.

To enrich the semantic features, we used the categorical value type associated with its frequency in the sentence; e.g., MPt_3, MP_2, MI_4. The association of the two types of values has improved the CRF model as compared to the usage of natural or binary values only. The generated sequence of numerical values disperses the model especially when the sentence bag of words also contains numbers.

In addition, we use the unigram technique combined with the POS information ([9, 12, 14]) to enrich the extraction of lexical features. The lexical features are represented by a bag-of-words and their corresponding POS.

##### Standardization of section headers

Structured abstracts do not follow a standard for the section headers. For example, different headers such as Population, Participants, Subjects, Method or Patients are used for the P element. We identified over 60 different headers within 310 structured abstracts. Using these headers in the training corpus could impair the effectiveness of the CRF learning model. To remedy this situation, we have used the most common headers proposed in [26], which are Objective, Method, Result and Conclusion, and we have extended the mapping list between these common headers and other headers (Table 7).

**Table 7:** Header mapping

Common header	Mapped header	Total
OBJECTIVE	AIM, OBJECTIVE, BACKGROUND AND OBJECTIVES, CONTEXT, …	37
METHOD	DESIGN, DESIGN AND METHODS, PATIENT(S), INTERVENTION, …	30
RESULTS	FINDINGS, MAIN RESULTS, OUTCOME MEASURES, …	13
CONCLUSION	CONCLUSION, DISCUSSION, IMPLICATIONS, SUMMARY, …	12

##### Grouping structural features

The structural features are simple to generate and are effective in training the model. Examples of those features are section header and sentence number. To our knowledge, these two features have not been reconciled before. We propose to align them as follows:

- If the sentence header is OBJECTIVE, then all the sentences in this section will have number 3; the number 3 is an arbitrary number close to the average size of the Objective section; its role is to standardize the structural feature.
- If the header of the sentence is METHOD, then all the phrases in this section will have number 6 that is an arbitrary number close to the average size of the METHOD section plus the average size of the OBJECTIVE section.
- If the header of the sentence is RESULT, then all the phrases in this section will have number 12.
- If the header of the sentence is CONCLUSION, then all the sentences in this section will have number 14.

The grouping promotes uniformity of features and consequently facilitates the training of the model and improves its accuracy. In fact, the features are processed as a bag of words in the CRF training; the more clustered these bag of words are, the better the CRF is supervised. For example, the following three combinations: (Objective 1), (Objective 2) and (Objective 3) will all be represented by the single combination (Objective 3).

##### Mixing structured and unstructured abstracts

Structured abstracts are favoured in the learning process of the CRF model, because they contain section headers that are an effective way to train the CRF model; for example, Population and Intervention elements tend to be in the Method section while the Outcome element is often placed in the Result section. However, in the training corpus, there are more unstructured abstracts than structured ones. Even more, most of Intervention and Population elements are in the unstructured abstracts of this corpus. To extract them correctly we should assign *heading section* to the sentences in unstructured abstracts, creating “pseudo” structured abstracts as follows:

- OBJECTIVE section for the sentences labeled “Background”;
- METHOD section for the sentences labeled “Population”, “Intervention” or “StudyDesign”;
- RESULT section for the sentences labeled “Outcome”;
- CONCLUSION section for the sentences labeled “Other”.

##### Other factors influencing the prediction

We had to reconcile the choice of the Gaussian prior with the quality of the training corpus. We set the Gaussian prior to 10 and introduced a redundancy indicating both the presence and absence of classes of interest: P, I and O.

### RBM stage

**Fig. 2:**
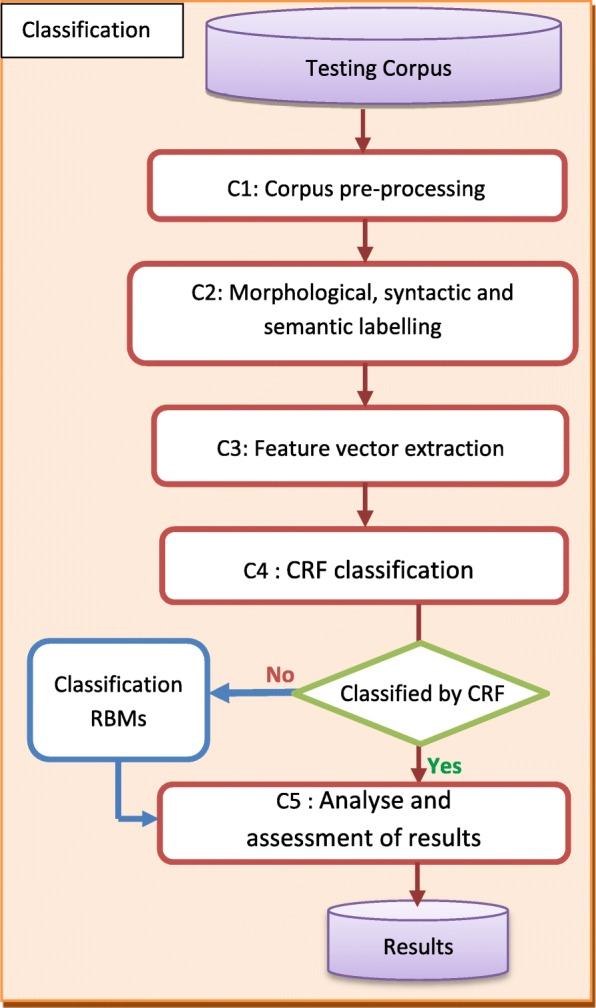
Incorporation of RBMs in the MLM classification process

RBMs are different from MLMs. MLMs are driven by a learning phase and probabilistic prediction at the sentence level (coarse-grained). RBMs can explore the semantics within the sentence (fine-grained) to extract PICO elements. RBMs can complement the MLMs to raise the accuracy above the thresholds (see 4.2 section). Figure 2 shows how we incorporate RBMs in the MLM classification process.

**Fig. 3:**
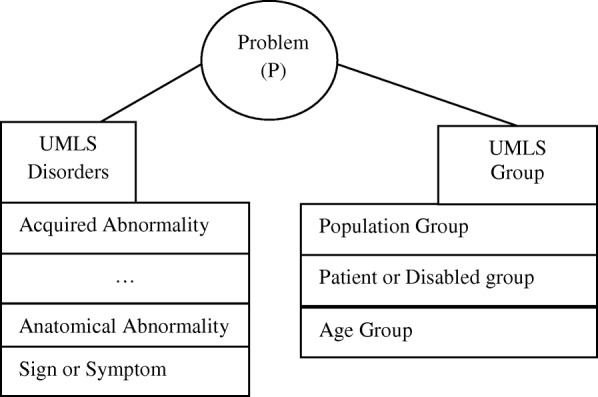
Conceptualization of the element P as a relationship between two UMLS groups: Disorders and Group

In our earlier research [27] we conceptualized the P element as a relationship between the semantic UMLS groups Disorders and Group [28] as shown in Fig. 3.

**Fig. 4:**
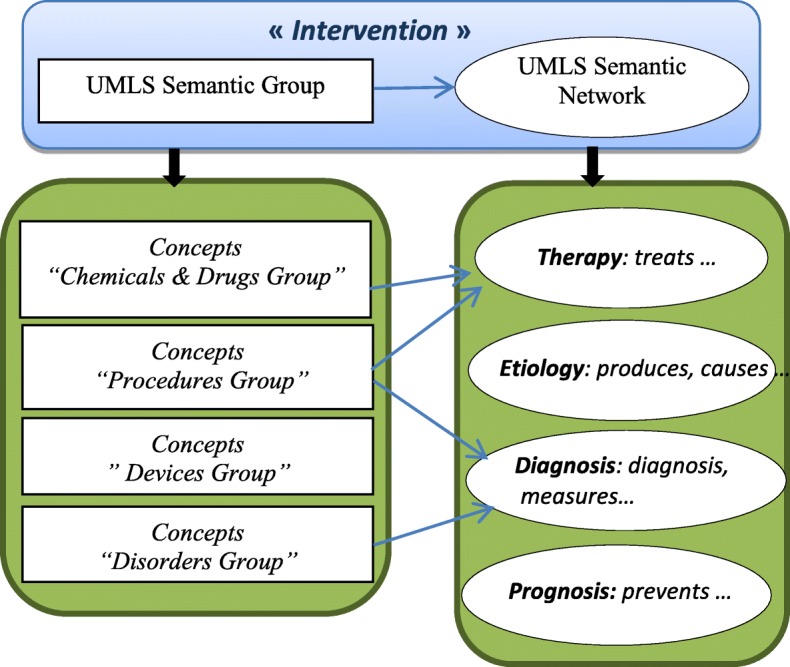
Conceptualization of the element I as a relation between UMLS semantic group and UMLS semantic network

In addition, we conceptualized the element ***I*** as a relation between UMLS Semantic Group and UMLS Sematic Network (Fig. 4).

We also specified RBM rules to extract the PICO elements; for example:

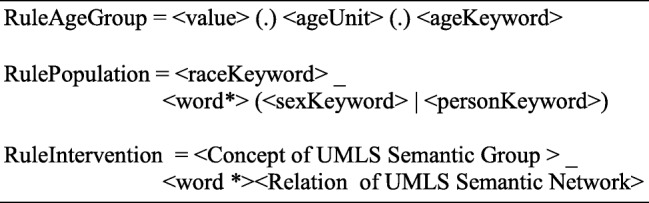



However, we found that these rules are not efficient; for example, the pattern <value> covers too many combinations. In addition, the pattern *<Relation of UMLS Semantic Network >* is ineffective (cTAKES covers only a few UMLS Semantic Relations) and the rule is too rigid (the pattern order creates an unnecessary dependency). We simplified these rules and based them on the MLM features to facilitate the integration of the two methods. Therefore, the rule that detects the presence of the P element is as follows:

f_1_> = 3 or f_2_> = 3 or.

(f_1_> = 1 or f_2_> = 1) and f_9_ = “METHOD” or.

f_2_> = 1 and f_9_ = “NOHEADING” and numSen < 4.

f_1_ and f_2_ are the semantic features of the MLM model and f_9_ is the positional feature (Table 4). Features f_1_ and f_2_ assess respectively the presence of the disorder part and the population part of the P element in the sentence, whereas f_9_ is the positional feature; when f_9_ is equal to the header METHOD, the constraint on f_1_ and f_2_ is relaxed. In fact, the P and I elements are usually in the section METHOD (Table 7). In the case of non-structured abstracts, the Intervention and Population elements are usually inside the first three sentences [8].

This rule is flexible; we could relax the constraint on f_1_ and f_2_ by choosing value smaller than 3. The value 3 represents the average value found in the sentence labelled as P in the MLM classification. For f_3_, the average was 2, but we kept the value 3 to enhance the quality of the results.

We specified the rule for the I element as follows:

f_3_ > = 3 or f_3_ > = 1 and f_9_ = “METHOD” or.

f_3_> = 1 and f_9_ = “NOHEADING” and numSen < 4.

## Results

Through the experiments, we were able to identify the best set of aspects (Table 8) that optimized the CRF model to reach the best F-scores (60% for the P element and 40% for the element I).

**Table 8:** Set of aspects that produced the best recall for P and I

Aspect	Best choice of aspect	Other assessed choices
Gaussian prior	10	0.1, 1, 10, 100
Model training-proportion	(100, 0%)	(50, 50%), (80, 20%), (90, 10%)
Training information layout	Standard	Information redundancy
Testing information layout	Redundant information	Standard
Mixing different features	All features	Part of them
Type of feature values	Categorical	Binary, natural
Grouping structural features	Yes	No

### Assessment of the CRF model

We applied the CRF model generated with the best choices of aspects on the test corpus of [12] in order to cross-validate our model against the results obtained in the Australian Language Technology Association (ALTA) Shared Task [12]. The test corpus consisted of 200 abstracts, 80 structured and 120 unstructured. The CRF model was applied in Mallet environment and the extracted PICO elements were evaluated according to the evaluation rules specified in phase C4 (Fig. 1). The best F-score was 60% for the P element and 40% for the element I. The CRF stage missed 73 of 200 abstracts in the case of the P element and 79 abstracts in the case of the I element. This is explained mainly by:

1. The CRF performed worse on the unstructured abstracts due to the structural features that are less precise in the unstructured abstracts than in the structured ones. In fact, the sentence header feature is always ‘NOHEADING’ in the case of an unstructured abstract, whereas it has a specific heading in the structured abstracts. In the missed abstracts for P, 21% of them are structured and 79% are unstructured. In the missed abstracts for I, 20% of them are structured and 80% are unstructured.
2. In the case of the I element, some of the missed abstracts are not related to therapeutic domain, but rather related to prognostic, etiologic and socio-medical researches.

In the ALTA shared task [12], ten different systems were evaluated using the same training and testing corpus. All these systems relied on MLM methods, namely Support Vector Machines (SVM), Stacked Logistic Regression, Maximum Entropy, Random Forests, and CRF. The reported F-scores of these systems vary from a lowest of 30% to a highest of 58% for the P and from a lowest 18% to a highest of 35% for the I.

In addition, we compared our results with the ones published in [11], the authors of this paper reported an F-score = 81% for the P element and a promising F-score = 81% for the I element.

**Fig. 5:**
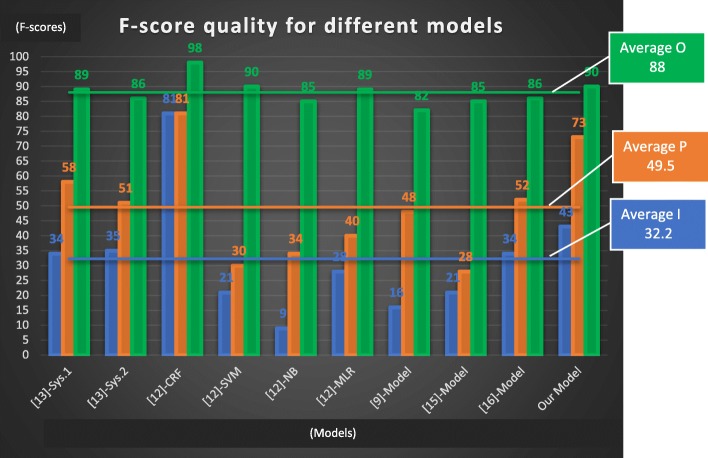
F-score quality for different models

We used ALTA training and test files. The comparison of the different models is based on the F-score because it compromises between the Precision and the Recall and because we do not always have the Precision and Recall from other researches. The distribution of PICO elements in the training corpus has a major impact on the F-score quality. For example, in the case of the P and I elements the training percentage is less than 7% and the average F-score of different models is of 49.75% for P and 32.43% for the I; whereas the training percentage of the O element is close to 36% and the average F-score is 88.28%. In this context, it is difficult to qualify a model by “good” or by “poor” based on a low percentage F-score threshold. For example, a model with F-score of 45% for the I element is not necessarily poor. However, we can compare the model quality against the average F-score for a sample of 10 models (Fig. 5).

Our model performed 1.47 better than the average for the P element, 1.33 better than the average for the I element and 1.02 better than the average for the O element. This is mainly attributed to the choice of our features; they are more exhaustive compared to [12], their standardization and harmonization contributed to make the CRF model learn efficiently.

Two other choices could affect our results:

- using cTAKES instead of MetaMap [29] as a tool for extracting UMLS concepts in a text,
- using CRF as a MLM algorithm.

More research is needed to assess the impact of these two elements on the results. In the case of the reference [11], the results may not be directly comparable. In fact, it is not clear to us if the results have been directly validated against the sentence labels provided in the test file. Based on the distribution of PIBOSO elements [11] and the number of abstracts used in the training phase (1000), it seems that the authors have used the sentences of the test file in the training phase, which could bias the results and set them higher. Nevertheless, the authors provided several results based on different techniques as shown in Table 9 and they reported F-scores of 81.32% for the P element and 81.06% for the I element.

**Table 9:** Comparison of our MLM results with the literature review results. Bold values show the best obtained F-scores

	P	I	O
Number of sentences in training (%)	662 (6.8)	565 (5.9)	3565 (36.6)
Our MLM stage - blind test corpus			
F-score	**73%**	**43%**	90%
The best F-scores in ALTA shared task [12]			
System 1	**58%**	34%	**89%**
System 2	51%	**35%**	86%
The best F-scores in paper [11]			
CRF	**81%**	**81%**	**98%**
SVM	31%	21%	90%
Nave Bayes	34%	10%	86%
Multinomial Logistic Regression	41%	28%	90%
Other papers F-score results using the same training and test corpora			
Kim et al. [14]	48%	16%	83%
Verbek et al. [30]	29%	21%	85%
Sarker et al. [31]	52%	34%	86%

We executed a 5-fold cross validation to assess overfitting and robustness of the model. In each set, we formed a subset (800 abstracts) for training and another subset (200 abstracts) as a blind test set. The 5-fold cross-validation sets shows that our model is stable and does not deviate dramatically from one fold to another.

During the validation of our results against the test file, we noticed a considerable number of abstracts in the test file that were not labeled with P or I (27 and 53% respectively) although some of them were a potential candidate for that.

Table 10 shows some examples of potential sentences for P and I elements, each sentence belonging to a different abstract. We thoroughly re-evaluated the results to exclude effects that may bias the results and underestimate the performance of our MLM model.

**Table 10:** Examples of potential sentences that are not considered in the test file of the ALTA shared task [12]

Examples of potential P sentences that are not considered in the test file	
“An estimated 20% of all breast cancer or ovarian and breast cancer cases have familial aggregation.” [32]	
“Clinical trials such as the Sudden Cardiac Death Heart Failure Trial (SCD-HeFT) are currently underway to investigate the role of the implantable defibrillator in patients with heart failure.” [33]	
Examples of potential I sentences that are not considered in the test file	
“Tizanidine hydrochloride is a very useful medication in patients suffering from spasticity caused by MS, acquired brain injury or spinal cord injury.” [34]	
“Here we describe the influence of local anesthesia and back-muscle-training therapy on subjective and objective pain parameters in 21 low-back-pain patients who had similar clinical status and neurophysiologic findings and whose recurrent low back pain.” [35]	
“Laparoscopy is highly accurate and effective in the management of peritoneal dialysis catheter dysfunction and results in prolongation of catheter life.” [36]	
“Here, vertebroplasty and kyphoplasty may provide immediate pain relief by minimally invasive fracture stabilisation.” [37]	

After re-evaluating the results, we were able to improve the F-scores of the P, I and O elements. The F-score increased from 73 to 79% for P, from 43 to 60% for I and from 90 to 92% for O.

### RBM stage results

We run the RBM stage on the missed abstracts by the CRF for P and I elements. Table 11 shows the results.

**Table 11:** RBM results on missed abstracts

	P	I
Unstructured abstract extraction	28 abstracts	28 abstracts
Structured abstract extraction	10 abstracts	14 abstracts
Missed	15 abstracts	7 abstracts
N/A (not applicable)	9 abstracts	55 abstracts
Total	62	104

The RBM stage was able to improve the overall results. Especially it performed well on the unstructured abstracts where the MLMs had failed. Most of N/A abstracts for the I element are not related to therapeutic domain; rather they treat an etiologic or prognostic domain and some of them are a socio-medical study. The number of N/A abstracts in the case of the P element is smaller, because the P element medical scope is larger in comparison with the I element. It can be related to an etiologic or prognostic domain or to a socio-medical study. If a disorder part or a demographic part of the P element is missing, the P extraction is partial.

The RBMs have a good recall rate and can complement the MLMs by improving the performance on the unstructured abstracts. However, the number of N/A (Not Applicable) abstracts affects their precision. The RBM coverage of the P and I elements is high so it has a tendency to label the N/A abstracts, which would lower the precision. The RBMs stage achieved an F-score of 66% for the P element and 55% for the I element.

In order to increase the RBM precision we should:

- Pre-filter the abstracts. In the context of these experiments the abstracts of the training and testing corpora were randomly sampled from the GEM [22] and AHRQ [23] institutions which explains the presence of the high number of the N/A abstracts for the I element. However, in the medical Question-Answer-System (QAS) context, the document filtering step of the QAS reduces the N/A abstracts; in fact, a filter is applied on the abstracts based on the question key words or the question type (therapy, etiology, prognosis, …).
- Tighten the constraints on the features f1, f2 and f3 in the RBM rules.

The good recall rate of the RBM rules allow them to back up the MLMs. Table 12 summarize our MLM, RBM and combined approach results, and compares them with those in the literature review.

**Table 12:** Results of MLM, RBM and combined approach

	Element P	Element I
Precision		
MLM	85%	65%
RBM	61%	40%
Combined (MLM & RBM)	77%	51%
Recall		
MLM	74%	57%
RBM	72%	86%
Combined (MLM & RBM)	83%	86%
F-score		
MLM with CRF	79%	60%
RBM	66%	55%
Combined (MLM & RBM)	**80%**	**64%**
ALTA [12] best F-scores		
MLM with CRF	**58%**	**35%**
Paper [11] best F-scores		
MLM with CRF	**81.3%**	**81.1%**

The MLMs and RBMs complement each other and the combined approach improves the F-scores of the P element from 79 to 80% and from 60 to 64% for the I element. We were able to match the F-score of reference [11] for the P element, but were not able to reach the same F-score for the I element, despite the improvement of the overall results. However, at this stage, the comparison with references [12] and [11] may not be relevant and legitimate anymore since the evaluation of the results is not necessarily based on the same criteria.

## Discussion

In this paper, we present a novel hybrid clinical NLP system using both MLMs and RBMs for PICO elements extraction. First, we evaluate the aspects involved in the creation of MLM model. Fine tuning these aspects helps us to improve the results despite the fact the corpus was not well balanced in terms of PICO element distribution. Next, we propose a set of RBM rules based on the MLM features. The performance achieved by our combined approach is competitive with previously published results. The MLM methods tend to be precise but they have a low rate of recall when the corpus is not representative or balanced. The RBMs support the MLMs in increasing the recall rate due to their efficiency with the unstructured abstracts where the MLMs failed to perform adequately.

We observed that the several PICO elements extraction projects do not rely on realistic environment setting; this is due mainly to the non-availability of representative training corpora. One of the avenues would be an agreement between researchers on constructing a representative and well-balanced PICO medical corpus like the ones used for Named-Entity recognition (NER), POS recognition and sentence recognition. This kind of corpus would foster a representative number (thousands) of sentences labelled by experts and require an agreement on the type of features, their specification, the tools and thesaurus to use for medical labelling, the type of extraction methods to use. In this context, our work could be of value in suggesting the features, the tool and the extraction methods to use.

We also observed that both P and O elements could overlap between primary question types as therapy, diagnosis, etiology or prognosis. However, the meaning of I element depends on the question type. In fact, the error rate for the I element classification is highly related to non-therapeutic abstracts. Training the I element with them creates a lot of noise in the learning process. Our team is currently investigating question type dependent models that are more effective for retrieving I elements.

We also noticed that the model reacts positively when we try to normalize the bags of words, for example, by grouping structural features, standardizing section headers, or using semantic features with categorical values. We think that it might be beneficial to do more work in analysing the model features to find bags of words that could be more efficient in the training phase.

In our study we did not take in consideration the impact of the tools used neither the choice of the ML methods (CRF vs other supervised algorithms); for example we do not know to which extent the usage of cTAKES instead of MetaMap would have affected the results.

## Conclusions

In this paper, we present a novel hybrid clinical NLP system using both MLMs and RBMs for PICO elements extraction. Our study of the PICO element extraction shows that the task is very challenging. The MLMs tend to have an acceptable precision rate but they have a low recall rate when the corpus is not representative. The RBMs backed up the MLMs to increase the recall rate and consequently the combination of the two methods gave better results. The performance achieved by our combined approach is competitive with previously published results.

## Abbreviations

AHRQ: Agency for Healthcare Research and Quality
ALTA: Australasian Language Technology Association
CEM: Clinical Element Models
CRF: Conditional Random Fields
cTAKES: Clinical Text Analysis and Knowledge Extraction System
EBM: Evidence-Based Medicine
FN: False Negatives
FP: False Positives
GEM: Global Evidence Mapping Initiatives
Mallet: MAchine Learning for LanguagE Toolkit
MLMs: Machine Learning Methods
NER: Named Entity Recognition
PIBOSO: “Problem, Intervention, Background, Study Design and Other”
PICO: “Problem, Intervention, Comparison and Outcome”
POS: Part-Of-Speech
QAS: Question-Answering System
RBMs: Rule Based Methods
RCT: Randomized Controlled Trial
SVM: Support Vector Machine
TN: True Negatives
TP: True Positives
UMLS: Unified Medical Language System
